# Development of Surgical Error Reduction System (SERS) for Laparoscopic Appendectomy by using Observational Human Reliability Analysis (OCHRA) model and to analyse its impact on patient outcomes

**DOI:** 10.29337/ijsp.181

**Published:** 2022-09-21

**Authors:** Girivasan Muthukumarasamy, Samer Zino, Benjie Tang, Pradeep Patil

**Affiliations:** 1 Department of General Surgery, Ninewells Hospital and School of Medicine, University of Dundee, Dundee DD1 9SY, UK; 2 Surgical Skills Centre, Dundee Institute for Healthcare Simulation, Ninewells, Hospital and Medical School, University of Dundee, Dundee, UK

**Keywords:** laparoscopic surgery, appendectomy, training, error analysis

## Abstract

This project is to develop a surgical error reduction system (SERS) for laparoscopic appendectomy by using observational Human Reliability Analysis (OCHRA) model and to analyse it impact on patient’s outcome.

## Background

Surgical removal of appendix is one of the commonest emergency surgical operations. Laparoscopic appendectomy has clear advantage compare to open in reducing post-operative pain, wound infections, length of hospital stay and return to normal activities [1]. In our institution all appendectomies are started laparoscopically and the conversion to open surgery is less than 5%. Laparoscopic appendectomy (LA) forms the index training procedure for general surgical trainees when they start emergency and minimally invasive surgeries. It is also the first operation the trainees start to do with minimal supervision. So, it is more likely to be associated with errors both consequential and inconsequential. Suboptimal surgical techniques and errors adversely affects the patient recovery and outcomes [2].

From previous and current work on laparoscopic cholecystectomy, bariatric surgery, eye surgery, and rectal cancer surgery, we have developed techniques for hierarchical task decomposition and analysis including human reliability analysis (HRA) which are applicable to laparoscopic procedures [2,3,4,5,6,7,8,9,10,11,12].

Since 1992, there has been has extensive educational and research experience in our centre. Studies have covered various areas including psychomotor skills assessment, ergonomics, task analysis and Human Reliability Analysis (HRA) in laparoscopic surgery. This has resulted in an established research unit with excellent resources for the proposed project. There is high definition video capture and processing facilities and local expertise in the field. Two authors carried out their higher degrees in the field of OCHRA and Psychomotor Skills assessment in Laparoscopic surgery.

## Aim

The aim of this project is to develop a surgical error reduction system (SERS) for laparoscopic appendectomy by using observational Human Reliability Analysis (OCHRA) model and to analyse it impact on patient’s outcome.

## Objective

### Primary

1. Develop a hierarchical task analysis (HTA) for Laparoscopic appendectomy.
2. Apply a standardised error measurement system to the HTA.
3. Correlate the errors with the immediate and long-term clinical outcomes.

### Secondary

1. To document for each task of the procedure, those elements that are essential, those that are optional, and those that ought to be avoided.
2. From this list, a ‘how to’ document would be compiled and distributed to trainees and junior surgeons.
3. To conduct personalised feedback to the participating surgeons and trainees, outlining areas of good, suboptimal and even dangerous performance.
4. Ultimately aiming to repeat this study in same cohort following feedback and period of training (with mentor and with laparoscopic trainer box).

## Methods

Local clinical governance approval will be obtained prior to the start of the prospective study. 100 consecutive laparoscopic appendectomy procedures will be recorded after obtaining informed consent from patients.

The study will be carried out in three phases: in phase one, a hierarchical task analysis (HTA) will be carried out by observing laparoscopic appendectomy procedures. This will be validated through a review by an expert panel comprising two laparoscopic consultant surgeons and an academic expert. The HTA will provide a framework for the second phase which will consist of the application of HRA to procedures performed by other surgeons and trainees in analysing the errors. Third phase is correlating the surgical errors with patient outcome and complications.

## Phase 1

### Categorisation of errors

We have developed a human error categorisation in order to describe and categorise the errors that might be observed in surgical operations, based on the external error modes found in SHERPA [2,3,4]. These external error modes are a categorisation of the various ways in which a physical procedural task may be erroneously performed. Underlying these observable errors is a variety of performance shaping factors (PSF’s) which must be sought separately from the operator. Note that in this context, “PSF” is being employed in a very narrow sense, to include only those factors that are observable in the video recordings. Whilst it is recognised that external PSF’s (such as operating theatre environment and experience of other theatre personnel) also plays a part in determining outcome, the assessment of such factors is not intended in the current study. The ergonomics of port position, instruments used, and special devices used like hemlocks, endo loops, staplers will all be observed and studied. Therefore, the post-operative questionnaire will be limited to those factors and events that occur during the course of the procedure, and (may) have a direct impact upon surgical performance. The development of this structured questionnaire will be an integral part of the study, the content dependent upon a pilot study of the first 10 cases. The questionnaire/proforma will be created for the operating surgeon/trainee to fill at the time of the operation (Appendix 1). The external error modes list forms a template to describe and categorise the observable errors in each step of the operation which will be the subject of this study. In order to reflect any motor control errors identified, a modified list of external error modes has been created to be used in the study (Table 1).

**Table 1:** External error modes.


1.	Step is ***not done***

2.	Step is ***partially*** completed

3.	Step is ***repeated***

4.	Second step is done ***in addition***

5.	Second step is done ***instead of*** first step

6.	Step is done ***out of sequence***

7.	Step is done with ***too much*** (speed, force, distance, time, rotation, depth)

8.	Step is done with ***too little*** (speed, force, distance, time, rotation, depth)

9.	Step is done in ***wrong*** (orientation, direction, point in space)

10.	Step is done on/with the ***wrong object***


### Error identification

The definition of error agreed at the Bellagio Conference on Human Error will be used for the present study, i.e., ‘*something that has been done which was*: *(i) not intended by the actor, (ii) not desired by a set of rules or an external observer, or (ii) that led the task or system outside acceptable limits*. The external expression of an error is its *consequence* which may be neutral, (inconsequential) or negative (consequential) [3,4,5,6,7]. This definition is similar to but more comprehensive to that proposed earlier by Swain.

Previously we considered any action (or omission) that resulted in a negative consequence, or increased the time of the procedure by necessitating a corrective action, fell outside the ‘*acceptable limits*’ and was therefore registered as a consequential error. We defined inconsequential error as action or omission that increased the likelihood of negative consequence and under slightly different circumstances could have had a consequential effect.

The details of error classification system were described in the pilot study. In brief, ten generic forms of error can be predicted with respect to the execution of a surgical task. These ten generic types (or external error modes), represent observed patterns of failure (Table 1) and fall in two categories in relation to the underlying causative mechanism. External error modes 1–6 correspond to the ability of the surgeon to execute the component steps in the correct order and hence these are collectively grouped as *procedural error modes*. In contrast external error modes 7–10 reflect manipulations with laparoscopic instruments by the surgeon to execute a specific component step of the operation and are categorized as *execution error modes*. This distinction is of practical importance because it determines the nature of prescriptive error-reduction system specific to the operation. Execution errors can be reduced by better training of operative skills and by improving instrument design; whereas procedural errors can be minimized by improving the knowledge (perhaps aided by drop-down menus) that ensure the correct choreography of execution, i.e., the surgeon performs the component tasks and steps of the operation in the correct order.

### Task analysis

Two individual consultant surgeons will carry out direct observational methodology of unedited videotapes. Prior to the study, they will receive training in human factors’ research by an accredited human factors specialist with an interest in surgical ergonomics and human factors and who is not involved in the operations [13,15].

The inter-rater consistency of the SERS system will be assessed by the consultant surgeon and human factors specialist in the initial pilot study.

It is acknowledged that not every event out with the task Analysis (blueprint) will necessarily lead to an adverse outcome, the labelling of such events as ‘errors’ allows this very aspect to be assessed. i.e. any event that falls out with the blueprint may be independently evaluated for subsequent negative impact on the procedure. All cases that result in postoperative complications or conversion will be reviewed by the expert panel.

The expert panel that includes consultant laparoscopic surgeons will provide consultation throughout the study and check the accuracy of the videotape analysis process.

A hierarchical task analysis will be undertaken on LA (n = 10). Footage will be collected and the best techniques and acceptable variations in technique are standardised. The data will be collected by video recording of the internal endoscopic view and assessing the proforma questionnaire filled in by the surgeon.

The task analysis will divide each component of the procedure into tasks and subtasks. The main components of laparoscopic appendectomy are outlined in Table 2. These will then be correlated with immediate procedural outcomes documented by the recordings of the internal view and the external manipulation. This will provide a framework to allow allocation of errors to different steps of the procedure, and identification of tasks and subtasks with different levels of effectiveness. The data obtained by the task analysis can be used to formulate a menu of the exact details of how the operation is done according to the preoperative and intra-operative assessment of the cases.

**Table 2:** Outline of hierarchical task analysis of Laparoscopic Appendicectomy.


TASKS	

1.	Insertion of ports and creation of pneumoperitoneum

2.	Diagnostic laparoscopy

3.	Identification of appendix, caecum and terminal ileum

4.	Mobilisation of appendix/caecum

5.	Division of mesoappendix or control of appendicular artery

6.	Secure the base of appendix

7.	Extraction of specimen

8.	Washout

9.	Removal of the ports

10.	Closure of the wound


## Phase 2

### Data collection and analysis of procedures

As for phase 1, video footage will be obtained from endoscopic views of the procedure. Each step of the LA as identified in phase 1 will be analysed and the observed errors categorised according to the list of external error modes (Table 1) combined with detailed factors that influenced task performance (Table 3) and recorded against the step in which they occurred.

**Table 3:** Dimensions within each error event of laparoscopic appendectomy.


1. Instruments used Instrument for retracting and exposure Dissecting instrument Instrument for haemostasis Instrument for tissue approximation

Comparing 5 and 10 mm cameras, camera in umbilical and LIF sites, hook versus other instruments, control of mesoappendix with diathermy or clips or hemlocks, control of appendix base with endoloops or hemlocks or staplers, extraction with or without bag, extraction through LIF or umbilical port

2. Steps involved preparatory steps Tool-tissue dynamics Tissue involved

3. Time taken (timestamped) from start of abdominal insufflation with CO_2_ — to clear abdominal view and safety check of port insertion. Diagnostic laparoscopy and confirmation of appendicitis and degree of appendicitis. Suction of fluid or pus and limited washout. Skeletonise appendix and control mesoappendix. Dissection of whole appendix and visualisation of base of appendix. Control of the appendix base. Safe extraction of appendix either in a retrieval bag or without bag. Total time

4. Probability of adverse event Observed High risk Medium risk Low risk

5. Consequence of technical error Bleeding Perforation Spillage of contents of appendix Infection Herniation

Other clinical consequence data collected are collections, need for antibiotics, post operative need for radiology, antibiotics and reintervention. This will lead to longer length of stay and readmissions.


The sample size (n = 100) for the study is determined by three considerations. First, it should adequately describe the type and frequency of errors enacted in the course of a LA. Secondly, it should demonstrate the influence of enacted errors on intra-procedural outcome and, if any, complications. Thirdly, it should be a large enough sample for the results to be seen as representative and reliable.

As it is impossible to pre-judge the results, precise sample size calculations cannot be performed.

Dissection techniques, errors and outcome data will be compared and analysed using t-test, p < 0.05 will be considered significant. Quantitative error data will be expressed as mean +/– SD (standard deviation).

## Phase 3

In addition to the enacted errors during each step of the operation, the following data will be collected for each operation:

- *Patient*: demographic data, height, weight, BMI, sex, previous abdominal surgery, known abdominal disease, medical condition (cardiac, respiratory and renal). Using these data, patient will be classified into high and low risk.
- *Appendicitis*: symptomatology and presentation, Uncomplicated or complicated
- *Laparoscopic surgeon*: level of training, years of experience and volume of work.
- *LA*: grade of difficulty as assessed by the surgeon, execution time, intra-procedural outcomes.
- *Outcome*: intra-operative and postoperative complications.

After each LA, each surgeon will be asked to reflect on their performance for each component of the operation, using a standardised generic checklist with detailed factors that influenced task performance (Table 3) to study the mechanisms underlying the observed errors (internal error modes). This will allow better understanding of the performance shaping factors [11,14].

Thus it will be possible to describe the nature and frequency of errors enacted during LA, and to correlate these errors with outcome in terms of intra-procedural outcomes, and complications. It will also be possible to identify the performance shaping factors that underlie the errors. Finally it will be possible to correlate both errors and PSFs with the level of experience and training of the laparoscopic surgeons.

In this way it should be possible to achieve the following:

1. To document for each task of the procedure, those elements that are essential, those that are optional, and those that ought to be avoided
2. From this list, a production of a “blueprint”/handbook would be compiled and distributed to trainees/junior surgeons
3. To identify error modes and error patterns committed by surgical trainees in comparison with expert surgeons to develop a better training in laparoscopic surgery to the trainees
4. Collaboration with other educational establishments:
  - Surgical College/training website
  - Linked in with Surgical Skills course – part of lap colorectal course
5. To conduct personalised feedback to the participating surgeons, outlining areas of good, suboptimal and even dangerous performance
6. To reinforce this with in-theatre reminders for additional LA cases
7. Ultimately aiming to repeat this study in same surgeons, following feedback (Outcomes 4 &5). Note: this will require additional study/grant/funding.

Our unit has done similar studies in the past and there is no direct cost involved in doing the study. The indirect cost is the time spent in analysing the video for errors and data collection. We estimate approximately two hundred hours will be needed to do the analysis and the study. All the authors are employed full time and contracted to do research work and studies.

Once we complete the study, we will publish the data and the results in open access surgical training and education journal. The study will help the surgical trainees to understand and improve error reduction. It will also standardise the competency and feedback assessment for doing laparoscopic appendicectomy. Ultimately the aim is to improve the patient care and outcome.

## Appendix 1

### Prospective study of Laparoscopic Appendectomy

Date of Operation and start time:

Patient CHI:

Patient consented for video recording: Y/N

Appendicitis confirmed pre-operatively by radiology – Y/N

Antibiotics commenced pre-operatively – Y/N

If no – Were antibiotics given at induction – Y/N

If not – Were antibiotics given intra-operatively – Y/N

Grade of Operating Surgeon (Cons/ST 1-8/Fellow) and First Assistant (Cons/ST 1–8/Fellow/F2)

Previous laparoscopic appendicectomy experience –

Operating Surgeon (<10, 10–25, 26–50, 51–100, >100), and

First Assistant (<10, 10–25, 26–50, 51–100, >100)

Was the consultant called for help – Y/N?

If yes – at what stage of the operation?

If consultant not scrubbed – was the consultant in theatre – Y/N

Urinary catheter inserted pre-operatively – Y/N or intraoperatively – Y/N

Port Number:

Port Sizes:

Size of laparoscope – 5mm/10mm

30degree laparoscope – Y/N

Abdominal access technique – Veress needle, Hassan, Visiport

Intra-abdominal pressure – 8,10,12mmHg

Camera port during dissection: – Umbilical port or LIF port

Local anaesthesia – type, volume, technique (Wound infiltration/TAP blocks)

Rectus sheath closure – J PDS/J Vicryl

Skin closure – Surgipro/Monocryl/Other

How many smoke extraction filters were used – 0,1,2

Duration of surgery – From start of skin incision to last suture (as precise as possible)

Post operative antibiotics plan

Signature:
